# Nuclear Cardiology in 2020 - Perspectives of the New SBC Guideline

**DOI:** 10.36660/abc.20190874

**Published:** 2020-02

**Authors:** Cláudio Tinoco Mesquita, Wilter dos Santos Ker, Jader Cunha de Azevedo

**Affiliations:** 1 Departamento de Radiologia - Hospital Universitário Antônio Pedro - Empresa Brasileira de Serviços Hospitalares - Universidade Federal Fluminense (EBSERH-UFF), Niterói, RJ - Brazil; 2 Hospital Pró-Cardíaco, Rio de Janeiro, RJ - Brazil; 3 Serviço de Medicina Nuclear - Hospital Universitário Clementino Fraga Filho-Universidade Federal do Rio de Janeiro (HUCFF-UFRJ), Rio de Janeiro, RJ - Brazil

**Keywords:** Coronary Artery Disease/diagnostic imaging, Myocardial Perfusion Imaging/methods, Prognosis, Biomedical Technology/trends, Positron Emission Tomography Computed Tomography/trends, Tomography Emission-Computed, Single/methods

To get to know, to discover, to communicate

François Arago

French physicist François Arago’s quote is one of the most powerful summaries of scientific activity, starting with the search for existing knowledge, followed by the discovery of new information and culminating in their prompt communication. This flow is essential for scientific progress to reach and transform society. Thus, in the medical area, guidelines are considered to be essential for the organization and guidance of conducts and knowledge in a structured manner and within a pre-established method. The development of consistent and up-to-date guidelines is one of the most important tasks of a medical specialty society and involves the considerable effort of multiple specialists in the field of knowledge, reviewers, layout editors, among others. In addition to the arduous and complex task, the preparation of recommendations has against it the uninterrupted flow of publications that appear every day and that can change the current state of knowledge. We should highlight the amplitude, up-to-date status, and extensive applicability of this Nuclear Cardiology Guideline^1^ jointly developed by the Nuclear Cardiology Area of the Department of Ergometry, Exercise, Nuclear Cardiology and Cardiovascular Rehabilitation (DERC), by the Department of Cardiovascular Imaging (DIC) of the Brazilian Society of Cardiology (SBC) and the Brazilian Society of Nuclear Medicine (SBMN).

In little over a decade, the management of chronic coronary artery disease (CAD) has undergone a paradigmatic shift towards an optimized clinical treatment, which consistently reduces the progression of atherosclerosis and prevents thrombosis and acute coronary syndrome.^2^ Myocardial revascularization is indicated in acute cases, in higher risk cases and in those whose symptoms are progressive or refractory to drug treatment.^3,4^ The SBC Nuclear Cardiology Guideline joins the Chronic Coronary Syndrome Guideline, reinforcing the importance of functional methods in the diagnosis of the etiology of symptoms in patients with suspected CAD, identifying the most at-risk patients, in therapeutic decision-making and in the follow-up of treatment response.^4^ The question whether myocardial revascularization should be the initial management strategy in patients with chronic CAD and moderate to severe ischemia^5^ seems to have been answered with the presentation of the ISCHEMIA Study, which showed no benefit from routine revascularization when added to optimized drug treatment.^6^ However, we highlight the role of revascularization in improving symptoms and quality of life, reinforcing the importance of shared and individualized decision-making in patients who remain symptomatic despite optimized clinical treatment. Notably, several situations that were excluded from the ISCHEMIA study are covered in detail in the text of the Nuclear Cardiology Guideline,^1^ such as patients with coronary trunk injury, recent acute coronary syndrome, angioplasty in the last 12 months, ejection fraction < 35% and those with progressive or unstable symptoms.

It is important to emphasize that Nuclear Cardiology is not restricted to the study of coronary disease only, but has undergone a revolution in recent years, with advances in equipment, software and tracers that make it important in the management of several conditions, for which the cardiologist previously had no tools to meet their needs. The Nuclear Cardiology Guideline^1^ takes a comprehensive and practical approach to this new application for the cardiologist. In Figure 1 we show some of the new applications in which nuclear cardiology has important practical significance.

**Figure 1:**
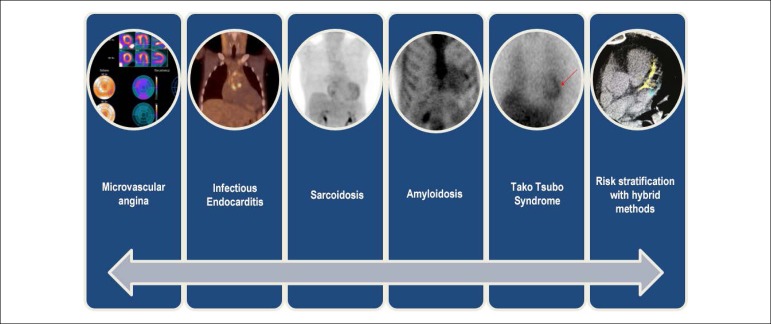
New applications of nuclear medicine in Cardiology where the use of the technique provides diagnostic, prognostic or guiding therapeutic decision-making information.

Among the new applications of nuclear cardiology, we highlight the use of 18F-FDG PET-CT and labeled leukocyte scintigraphy, as they were the nuclear medicine techniques included in the international algorithms and consensuses of investigation of infectious endocarditis in valve prostheses and in cases of suspected infection in implantable devices, such as pacemakers and defibrillators.^7^ The SBC Nuclear Cardiology Guideline addresses in detail the basis of the use of these techniques in modern cardiology practice.

Another important new recommendation for the use of 18-FDG PET-CT included in the guideline is cardiac sarcoidosis. In addition to a vital contribution to the diagnosis of cardiac sarcoidosis,^8^ PET-CT is crucial for monitoring treatment response, and its serial use is recommended to guide the use of immunosuppressants and anti-inflammatory drugs.^9^

Nuclear cardiology has become important in the diagnosis of cardiac amyloidosis caused by transthyretin deposits. A positive result in a scintigraphy with bone tracers, such as 99m-Technetium pyrophosphate, in the absence of light chain screening in blood and urine, allows the diagnosis of cardiac amyloidosis by transthyretin and correlates with the cardiac biopsy, which may prevent the latter. With the development of treatments that delay the deposition of transthyretin protein in the heart and reduce mortality and morbidity, myocardial pyrophosphate scintigraphy has gained additional relevance.^10,11^

The use of 123I-MIBG cardiac scintigraphy is based on the unique opportunity to evaluate the autonomous sympathetic component of cardiac innervation. The adrenergic impairment identified with this technique allows early detection of cardiotoxicity related to cancer treatment, stratifying the risk of sudden death in patients with heart failure^12^ and assisting in the diagnosis of Tako-Tsubo Syndrome.^13^

A modern and evolving chapter of Nuclear Cardiology, which is addressed in the SBC Guideline, is the assessment of microcirculation. Data from the Core 320 study and the ISCHEMIA study itself confirmed that a significant number of patients have angina and ischemia in the absence of coronary obstruction.^14^ The evaluation of these patients using PET-CT techniques allowed us to identify the presence of microvascular ischemia as responsible for most cases, which implies an adverse prognosis and specific treatment.^15^ The flow reserve assessment through PET-CT is the most appropriate technique to investigate these cases and is recommended in international guidelines and in the SBC Guideline. With the rapid advancement of high-performance machines with solid CZT detectors and improved software, the new SPECT chambers allow high-quality images with low radiation exposure and will contribute to the evaluation of these cases with studies demonstrating their validation, in comparison to the PET-CT equipment.^16^ The recognition of microvascular angina reinforces the importance of functional techniques and that a CAD assessment focused on the anatomy of CAD may lead to the underdiagnosis in cases of microvascular angina and overtreatment in cases where anatomic lesions do not have a functional significance.

One last part to be highlighted is the intersection between the several imaging modalities with hybrid equipment and software that allow the collection and analysis of nuclear cardiology data concomitantly with computed tomography or magnetic resonance imaging. The integration of exam information from different modalities into SPECT-CT, PET-CT and PET-MR equipment enhances the amount and quality of available information for cardiologists to make decisions in patient management. Even the integration of information from exams acquired from separate equipment can increase the potential for risk stratification and improve patient management.^17^ Ongoing studies will allow better definition of which patient groups will routinely benefit from these strategies.

In conclusion, cardiology has come a long way in recent years and so has nuclear cardiology. The new nuclear cardiology guideline by SBC enables us to learn about the most significant findings and publications through structured recommendations that impact the practice of modern cardiology.

## References

[1] Mastrocola LE, Amorim BJ, Vitola JV, Brandão SCS, Grossman GB, Lima RSL (2020). Atualização da Diretriz Brasileira de Cardiologia Nuclear - 2020. Arq Bras Cardiol.

[2] Stone GW, Hochman JS, Williams DO, Boden WE, Ferguson TB, Harrington RA (2016). Medical therapy with versus without revascularization in stable patients with moderate and severe ischemia the case for community equipoise. J Am Coll Cardiol.

[3] Boden W, O'Rourke R, Teo K, Hartigan, Maron D, Kostuk E (2007). Optimal medical therapy with or without PCI for stable coronary disease. N Engl J Med.

[4] Knuuti J, Wijns W, Saraste A, Capodanno D, Barbato E, Funck-Brentano C (2019). 2019 ESC Guidelines for the diagnosis and management of chronic coronary syndromes. Eur Heart J.

[5] Reynolds HR, Picard MH, Hochman JS (2015). Does ischemia burden in stable coronary artery disease effectively identify revascularization candidates. Circ Cardiovasc Imaging.

[6] Maron DJ, Hochman JS, Brien SMO, Reynolds R, Boden WE, Stone GW (2018). International Study of Comparative Health Effectiveness with Medical and Invasive Approaches (ISCHEMIA) trial: rationale and design. Am Heart J.

[7] Habib G, Lancelloti P, Antunes M, Bongiorni MG, Casalta JP, Del Zotti (2015). Guidelines for the management of infective endocarditis. Eur Heart J.

[8] Bois JP, Muser D, Chareonthaitawee P (2019). PET/CT Evaluation of Cardiac Sarcoidosis. PET Clin.

[9] Ramirez R, Trivieri M, Fayad ZA, Ahmadi A, Narula J, Argulian E (2019). Advanced imaging in cardiac sarcoidosis. J Nucl Med.

[10] Gillmore JD, Maurer MS, Falk RH, Merlini G, Damy T, Dispenzieri A (2016). Nonbiopsy diagnosis of cardiac transthyretin amyloidosis. Circulation.

[11] Treglia G, Glaudemans AWJM, Bertagna F, Hazenbeg BPC, Erba P, Giubbini R (2018). Diagnostic accuracy of bone scintigraphy in the assessment of cardiac transthyretin-related amyloidosis: a bivariate meta-analysis. Eur J Nucl Med Mol Imaging.

[12] Narula J, Gerson M, Thomas GS, Cerqueira MD, Jacobson AF (2015). (123)I-MIBG imaging for prediction of mortality and potentially fatal events in heart failure: The ADMIRE-HFX Study. J Nucl Med.

[13] Sabra MMM, Costa FS, de Azevedo JC, Mesquita CT, Verberne HJ (2019). Myocardial perfusion scintigraphy during chest pain: An atypical presentation of takotsubo cardiomyopathy?. J Nucl Cardiol.

[14] Schuijf JD, Matheson MB, Ostovaneh MSMR (2019). Ischemia and no obstructive stenosis (INOCA) at CT angiography, ct myocardial perfusion, invasive coronary. Radiology.

[15] Bairey Merz CN, Pepine CJ, Walsh MN, Fleg JL (2017). Ischemia and no obstructive coronary artery disease (INOCA): developing evidence-based therapies and research agenda for the next decade. Circulation.

[16] Agostini D, Roule V, Nganoa C, Roth N, Baavour R, Parienti JJ (2018). First validation of myocardial flow reserve assessed by dynamic 99mTc-sestamibi CZT-SPECT camera: head to head comparison with 15O-water PET and fractional flow reserve in patients with suspected coronary artery disease. The WATERDAY study. Eur J Nucl Med Mol Imaging.

[17] Siqueira FPR, Mesquita CT, Santos AAS, Nacif MS (2016). Relationship between calcium score and myocardial scintigraphy in the diagnosis of coronary disease. Arq Bras Cardiol.

